# Transcranial Near-Infrared Laser Transmission (NILT) Profiles (800 nm): Systematic Comparison in Four Common Research Species

**DOI:** 10.1371/journal.pone.0127580

**Published:** 2015-06-03

**Authors:** Paul A. Lapchak, Paul D. Boitano, Pramod V. Butte, David J. Fisher, Thilo Hölscher, Eric J. Ley, Miriam Nuño, Arne H. Voie, Padmesh S. Rajput

**Affiliations:** 1 Department of Neurology, Cedars-Sinai Medical Center, Los Angeles, California, United States of America; 2 Department of Neurosurgery, Cedars-Sinai Medical center, Los Angeles, California, United States of America; 3 BURL Concepts Inc., San Diego, California, United States of America; 4 Department of Surgery, Cedars-Sinai Medical center, Los Angeles, California, United States of America; Massachusetts General Hospital, UNITED STATES

## Abstract

**Background and Purpose:**

Transcranial near-infrared laser therapy (TLT) is a promising and novel method to promote neuroprotection and clinical improvement in both acute and chronic neurodegenerative diseases such as acute ischemic stroke (AIS), traumatic brain injury (TBI), and Alzheimer’s disease (AD) patients based upon efficacy in translational animal models. However, there is limited information in the peer-reviewed literature pertaining to transcranial near-infrared laser transmission (NILT) profiles in various species. Thus, in the present study we systematically evaluated NILT characteristics through the skull of 4 different species: mouse, rat, rabbit and human.

**Results:**

Using dehydrated skulls from 3 animal species, using a wavelength of 800nm and a surface power density of 700 mW/cm2, NILT decreased from 40.10% (mouse) to 21.24% (rat) to 11.36% (rabbit) as skull thickness measured at bregma increased from 0.44 mm in mouse to 0.83 mm in rat and then 2.11 mm in rabbit. NILT also significantly increased (p<0.05) when animal skulls were hydrated (i.e. compared to dehydrated); but there was no measurable change in thickness due to hydration.

In human calvaria, where mean thickness ranged from 7.19 mm at bregma to 5.91 mm in the parietal skull, only 4.18% and 4.24% of applied near-infrared light was transmitted through the skull. There was a slight (9.2-13.4%), but insignificant effect of hydration state on NILT transmission of human skulls, but there was a significant positive correlation between NILT and thickness at bregma and parietal skull, in both hydrated and dehydrated states.

**Conclusion:**

This is the first systematic study to demonstrate differential NILT through the skulls of 4 different species; with an inverse relationship between NILT and skull thickness. With animal skulls, transmission profiles are dependent upon the hydration state of the skull, with significantly greater penetration through hydrated skulls compared to dehydrated skulls. Using human skulls, we demonstrate a significant correlation between thickness and penetration, but there was no correlation with skull density. The results suggest that TLT should be optimized in animals using novel approaches incorporating human skull characteristics, because of significant variance of NILT profiles directly related to skull thickness.

## Introduction

Transcranial near-infrared laser therapy (TLT) is a promising and novel method to promote neuroprotection and clinical improvement in acute and chronic neurodegenerative diseases such as acute ischemic stroke (AIS), traumatic brain injury (TBI), Alzheimer’s disease (AD), Parkinson’s disease (PD) [1–14]. Of all the possible indications noted above, TLT for stroke is the most advanced, having been studied in 3 clinical trials [15–18], but demonstrating reproducible TLT efficacy in a diverse worldwide patient population has been problematic. Significant and important translational and clinical advances have also been made in the area of TBI treatment by Hamblin and colleagues [3, 8, 19–23].

TLT has been developed as a possible method to offer stroke patients an alternative, efficacious treatment to promote the recovery of clinical function following an ischemic stroke (reviewed in [3, 4, 24–26]), promote their well-being and allow stroke patients to maintain living independence. Studies with continuous wave (CW) TLT using a power density of 7.5 mW/cm^2^ [27], showed that TLT was effective on a behavioral endpoint when applied 24 hours after ischemia, but not before. DeTaboada et al. [28] published similar findings using CW TLT (power density 7.5 mW/cm^2^). Lapchak and colleagues used a rabbit small clot embolic stroke model to determine the therapeutic window for CW TLT [13] and showed a beneficial effect of CW NILT (power density of 10–25 mW/cm^2^) on behavior when applied up to 6 hours following a stroke. Despite the efficacy of low dose TLT, Ilic et al. [29] showed that there is a limit of CW TLT that can be beneficial without resulting in pathological changes (i.e. tissue necrosis) and behavioral deficits. This should be taken into consideration when developing TLT to treat brain disease.

Based upon efficacy in 2 species, 3 NeuroThera Effectiveness and Safety Trial (NEST) clinical trials [15–17] were mounted consecutively to evaluate the efficacy of CW TLT [15–17, 26] in stroke patients. In the 3 randomized and blinded trials, NEST-1 [15], NEST-2 [16, 17] and NEST-3 [18], continuous wave CW TLT was used within approximately 24 hours of an ischemic stroke, although this was variable amongst the 3 trials. While there was an efficacy signal in NEST-1 (clinical function measured using the modified Rankin Scale and National Institutes of Health Stroke Scale) and in a subpopulation of NEST-2 stroke trial patients, there was no effect of NILT on infarct volume in NEST-2[17], and NEST-3 was halted due to futility, because of a lack of efficacy [18].

There are numerous possible reasons that could contribute to the lack of reproducible, robust efficacy in the NEST trials that need to be delved into so that TLT can one day be used effectively. First, translational studies of the CW TLT NeuroThera device did not adhere to STAIR guidelines during development [30, 31]. Secondly, and most important, there is no evidence demonstrating that the treatment was adequately optimized prior to NEST trial initiation. While there is brief abstract report in the scientific meeting literature [32] discussing NILT, there is limited peer-reviewed literature documenting optimized parameters for NILT across the skull into the brain tissue that can be used as a guide for clinical trial design. For example, the original NEST-1 trial article by Lampl and colleagues specified a cortex surface fluence of 1 J/cm^2^ [15], the NEST-2 trial paper by Zivin et al. does not specify any power density treatment regimen [16], and the NEST-3 trial article is also devoid of important information regarding the treatment regimen used in the study [18].

We hypothesize that the failure of TLT in the NEST-3 clinical trial patient population may be due to TLT penetration variability and inadequate optimization of the NILT treatment regimen used in AIS patients [15, 16, 18]. The differential effects of TLT in clinical trials versus translational models suggest that the NILT parameters may have been insufficient to promote neuroprotection and clinical improvement in a diverse population of stroke patient within the extensive enrollment period (24 hours). It is possible that the power density did not adequately penetrate the human skull to activate biological mechanisms postulated to be important for neuroprotection and functional recovery (reviewed in [3, 4, 24–26]). Since we hypothesize that CW TLT treatment regimen may not have been optimal, we designed this systematic multiple species study to precisely measure NILT penetration through the skull to further understand NILT dynamics.

## Methods

### NILT measurements

**Fig 1:**
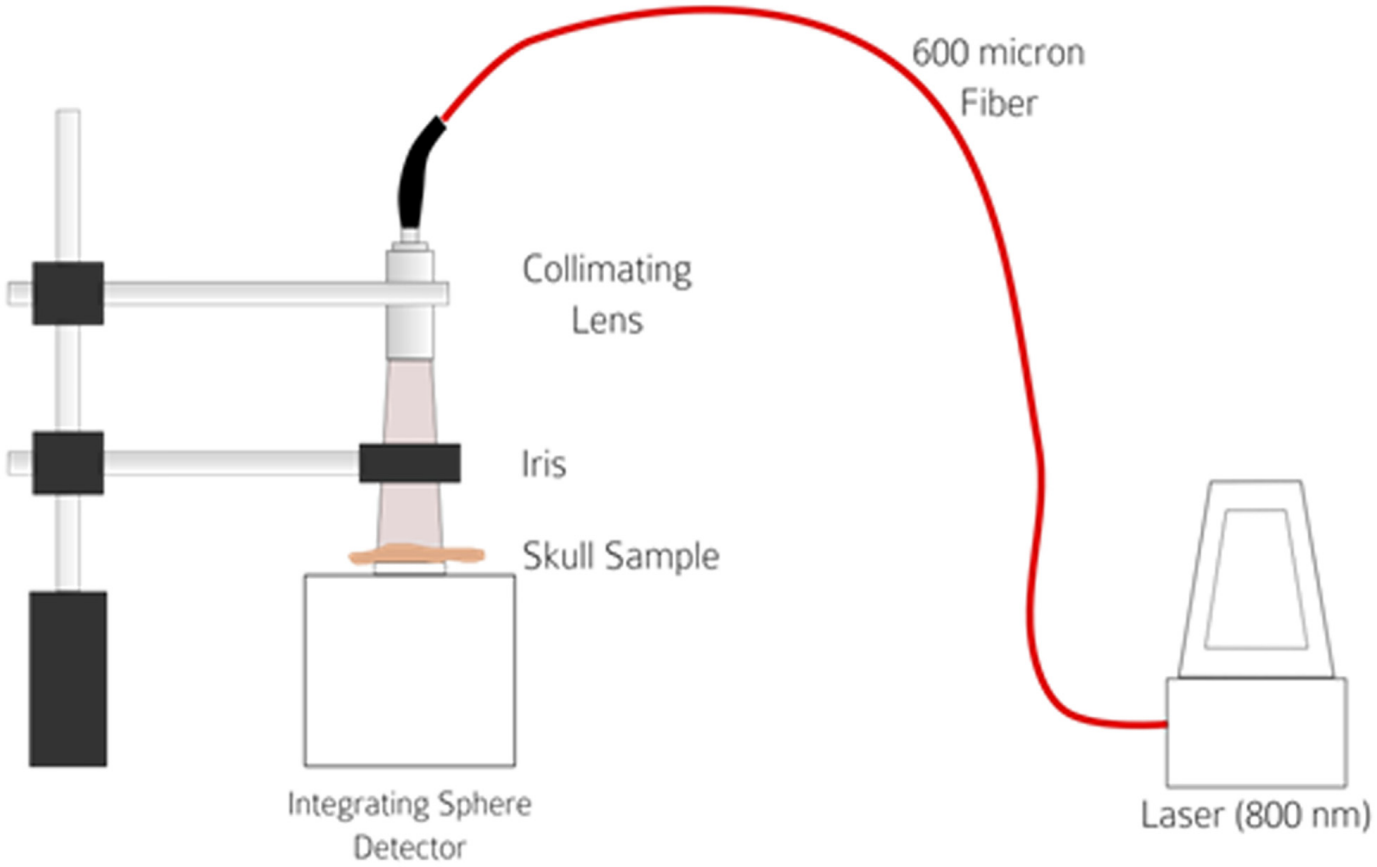
NILT Penetration Physical Set-up. Schematic diagram of the experimental setup used in measuring the light penetration of skulls from various species.

**Fig 2:**
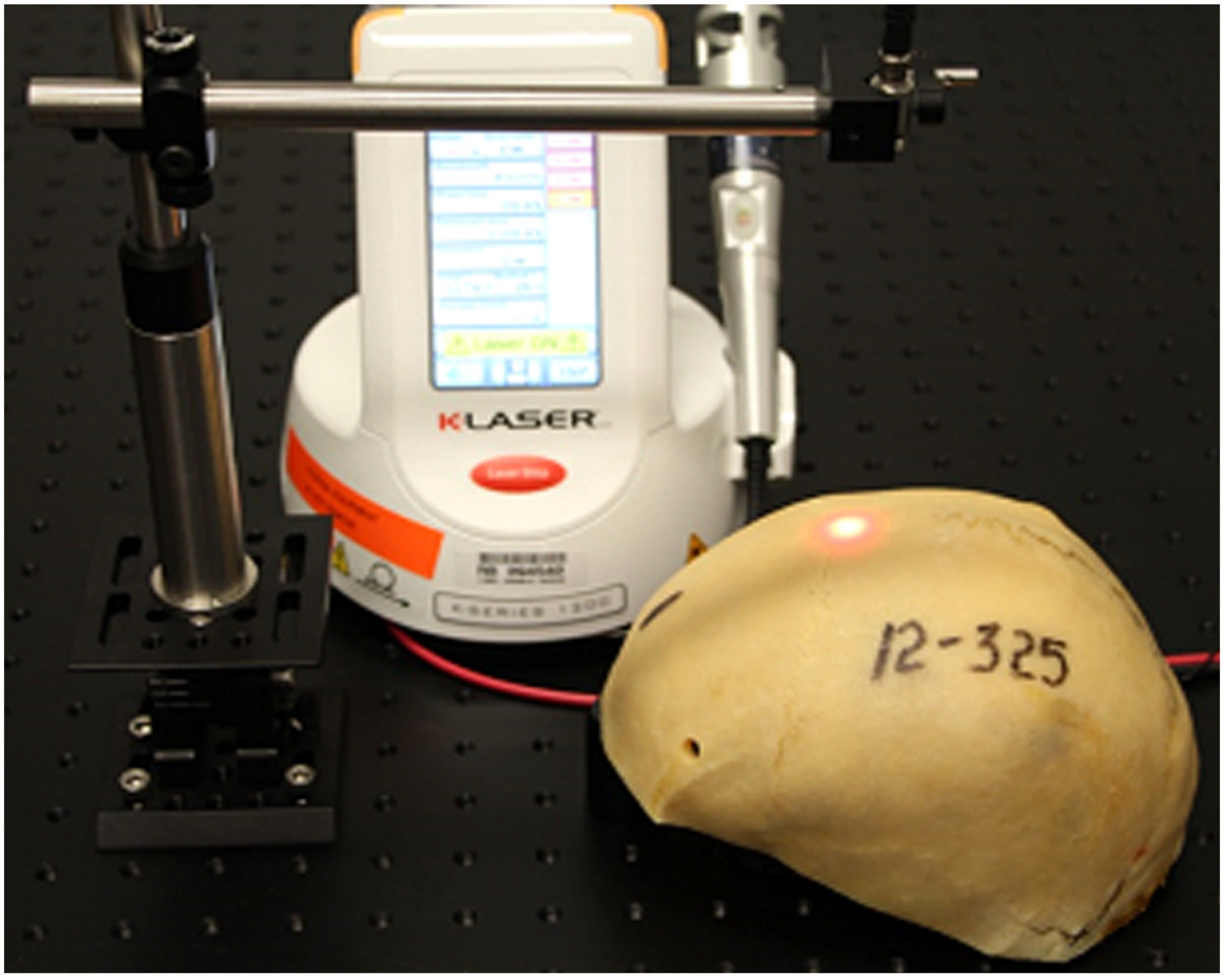
NILT Penetration Physical Set-up. Penetration Assembly and NILT Treatment at Bregma for example human calvaria #12–325.

**Fig 3:**
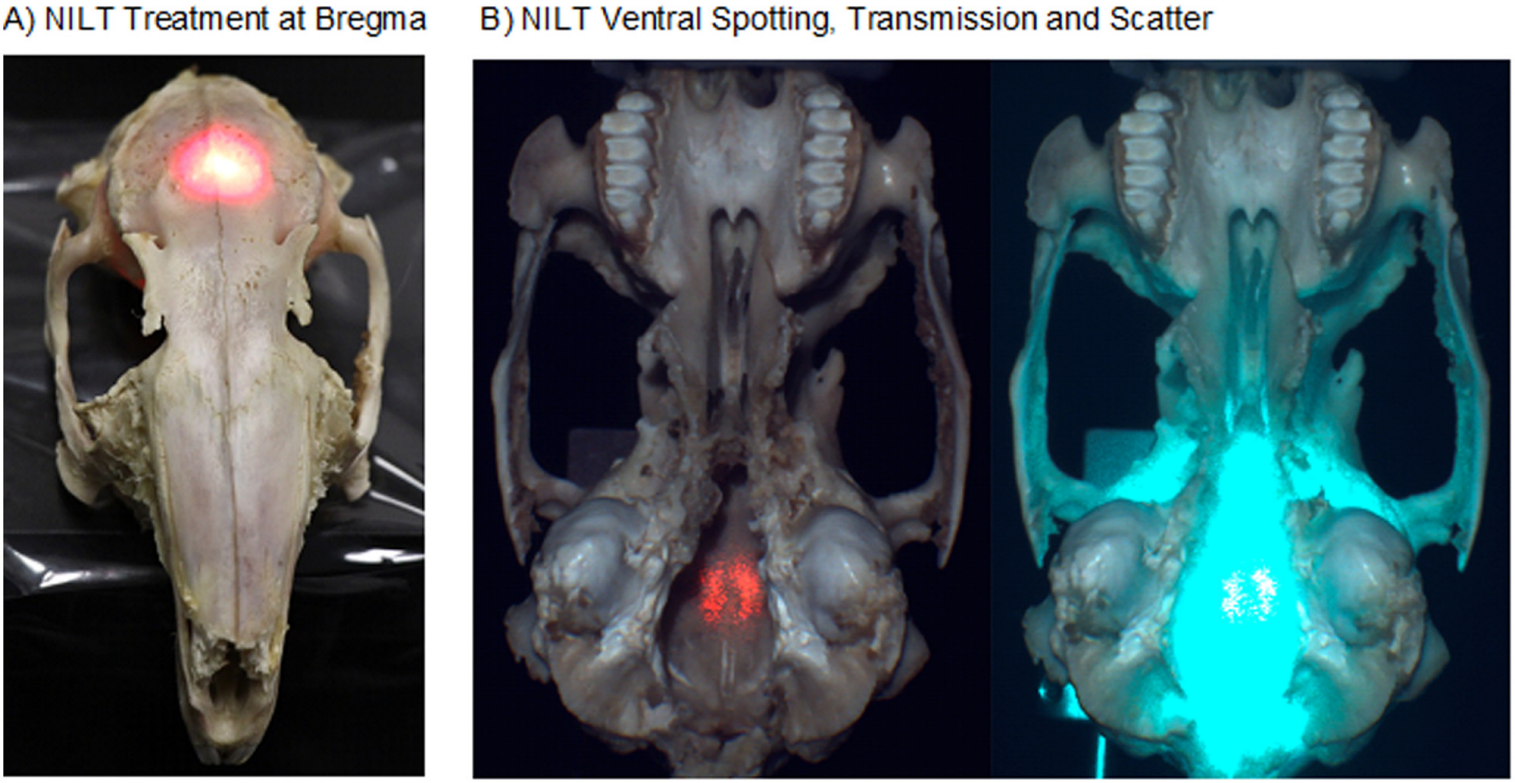
Dehydrated Rabbit Skull NILT Transmission. (A) NILT Treatment at Bregma showing the laser spotting light, (B) NILT Penetration and Scatter- Ventral surface of the rabbit skull showing the NILT penetration profile (A) and extensive diffusion pattern observed (B).

**Fig 4:**
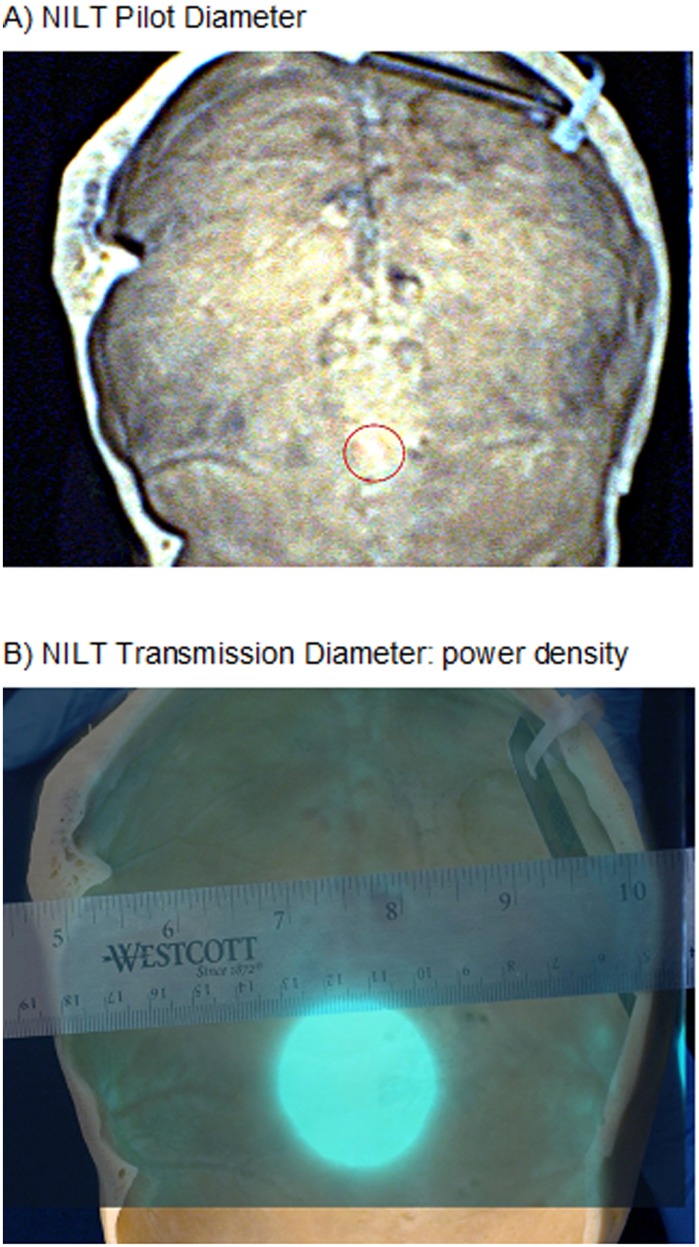
Dehydrated Human Calvaria #12–325 NILT Penetration. Ventral surface of the human skull showing NILT transmission (A) and the limited diffusion pattern observed (B).

For the studies, we used a K-Laser USA LLC (Franklin, TN, USA) model K-1200 dual wavelength (800 nm and 970 nm) Class IV laser with variable power output in the 800 nm mode to reproduce the regimen used in preclinical, translational and clinical studies (Fig 1). We used 3 different power densities from 200–700 mW/cm^2^ to determine NILT penetration profiles through the skulls/calvaria as a function of power density. The laser light was coupled to a 3 meter, 600 micron fiber (FT600UMT, NA 0.39, Thorlabs Inc, Newton, NJ, USA) which was connected to collimator (F220SMA-780, 780 nm, f = 11.07 mm, NA = 0.26, Thorlabs). The distance from the lens was adjusted in order to get 11.7 mm diameter beam. This gave an area of 107.5 mm^2^ (1.075 cm^2^), thus in order to achieve power densities of 200-700mW/cm^2^ for rat, rabbit and human (Figs 2–4). For mouse skulls, an area of 2.5mm was utilized. The laser power was detected using an Integrating Sphere Photodiode Power Sensor (S142C, Thorlabs Inc, Newton, NJ, USA).

### Skull & Calvaria sources

**Fig 5:**
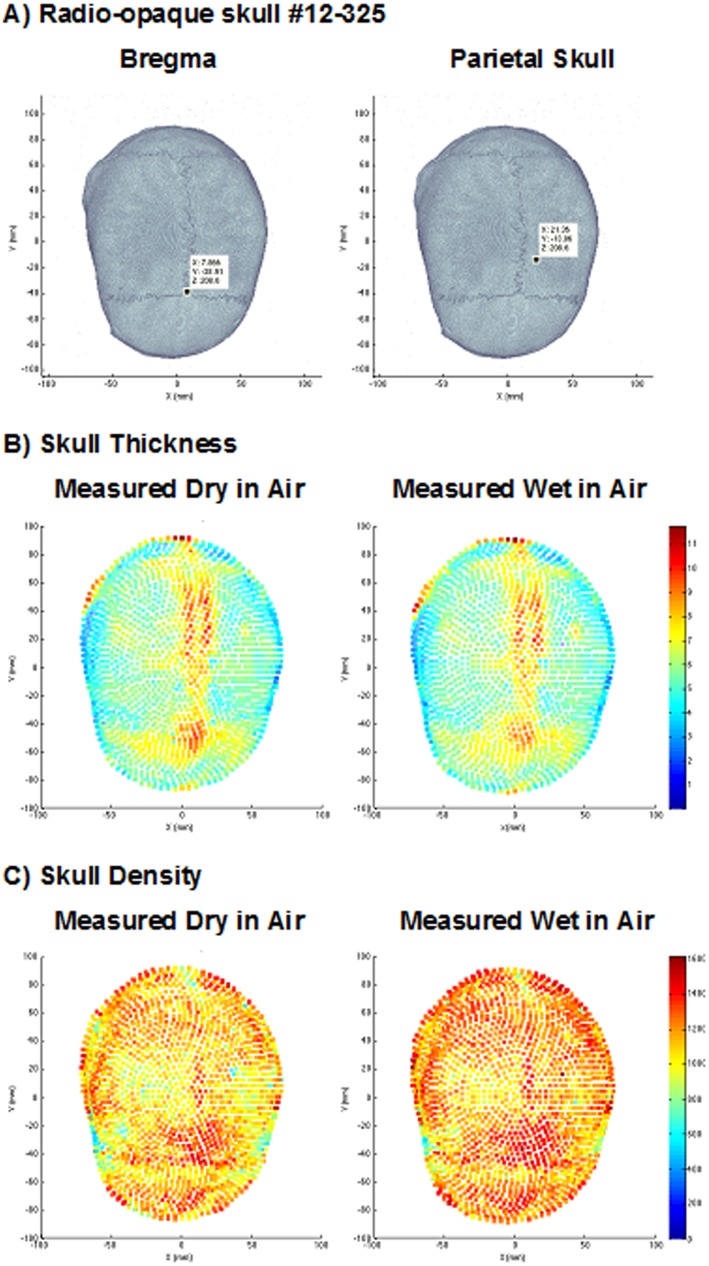
CT Scan Analysis of Calvaria #12–325. (A) Radio-opaque skull- Human Calvaria Measurement Point coordinates; (B) Skull Thickness Profiles and (C) Skull Density Profiles. Parametric maps of overall skull thickness and density. Vector positions for measurements are shown in A, Thickness (B) and density (C). Left panels dehydrated calvaria measurement in air; Right panels hydrated degassed measured in air.

For this study, all animal skulls were obtained from animals purchased under Cedars-Sinai Medical Center Institutional Animal Care and Use Committee (IACUC) approved protocols, which were conducted in accordance with the United States Public Health Service's Policy on Humane Care and Use of Laboratory Animals. Mouse skulls were obtained from 12-week old male C57BL/6 wild type mice (strain #000664) obtained from Jackson Laboratory (Bar Harbor, Maine). Rat skulls were obtained from 12–16 week old male Sprague-Dawley rats obtained from Harlan Inc., Hayward, CA. *Oryctolagus cuniculus* rabbit skulls were obtained from 10–14 week old male New Zealand white rabbits obtained from L.F.P.S Inc. (Norco, CA). Skulls were obtained from animals purchased under Cedars-Sinai Medical Center IACUC-approved stroke and traumatic brain injury protocols #2620, #4089 and #3231, respectively. Since skulls were removed post-mortem (i.e. after euthanasia), specific IACUC approval was deemed unnecessary for skull use in this study. Human skulls were obtained from the University of California San Diego, Department of Anatomy skull bank. Each skull was UCSD-tagged with an inventory number, sex and age All human skull information has been previously documented by Voie et al. [33]. Throughout the manuscript, skull #12–325 data is highlighted since it was used in the photographic series in Figs 2, 4 and 5.

### Skull thickness measurements

For animal skull thickness, we used a Mitutoyo Ratchet Digimatic Micrometer MDC-1”PJ with 0.00127mm precision. According to the certificate of inspection, the uncertainty of measurement is U = 0.000044 inches. Human skull thickness and density were measured using the methods developed and described in detail by Voie et al.[33]. Briefly, the skull was placed in the bore of a Discovery CT750 HD (GE Medical Systems, Waukesha, WI, USA) CT scanner and scanned to create a set of DICOM images (OsiriX, OsiriX Foundation, Geneva, Switzerland), which were read into MATLAB (The MathWorks Inc., Natick, MA, USA) to create 3D matrix of CT values, along with the voxel coordinates. Parametric skull maps are presented to demonstrate how each skull has a unique spatial distribution of both thickness and density (Fig 5). The heterogeneity of thickness in an individual calvarium is central to NILT penetration.

### Photographic Images

Photos were taken using a Canon EOS Rebel T3 black (Melville, NY, USA). The color-enhanced NILT diffusion pattern through the rabbit and human skulls were captured using a JAI (AD130 GE) camera with a capability of simultaneously capturing both near-IR and visible light images. The lens used for capturing the image was a 16 mm lens from Edmund optics (Techspec VIS-NIR fixed focal length).

### Statistical & Correlation Analysis

Descriptive statistics such as means and standard error mean (SEM) are reported throughout. Correlation (Pearson) and statistical analysis was done using Statistical Analysis Software (SAS, SAS Institute Inc., Cary, NC, USA). NILT penetration comparisons between dehydrated-and hydrated skulls done using an unpaired t-test (Graph Pad Inc.).

## Results

In the present study, we systematically measured NILT through the skull of 4 different species, including mouse, rat, rabbit and human to determine if the penetration profiles differ across species.

Fig 1 is a diagrammatic representation of the physical set-up used to study NILT profiles using the K-Laser device and Figs 2 and 3 show the K-Laser device measuring NILT through human skull #12–325. Note the red spotting beam on the surface of the skull at bregma.

Fig 3 shows an example of a rabbit skull used for NILT analysis. In panel A, we show the red spotting beam on the surface of the skull at bregma. In panel B, we show (left) laser penetration (i.e.: transmission) through the skull using the red spotting beam and (right) extensive diffusion pattern of laser light measured on the ventral surface of the skull. In comparison, Fig 4 shows the area of laser penetration (i.e. transmission) through the human calvaria (red circle) and (B) limited scatter pattern of light transmission through the calvaria.

### Skull Penetration Studies

#### Animal Skull Studies

**Fig 6:**
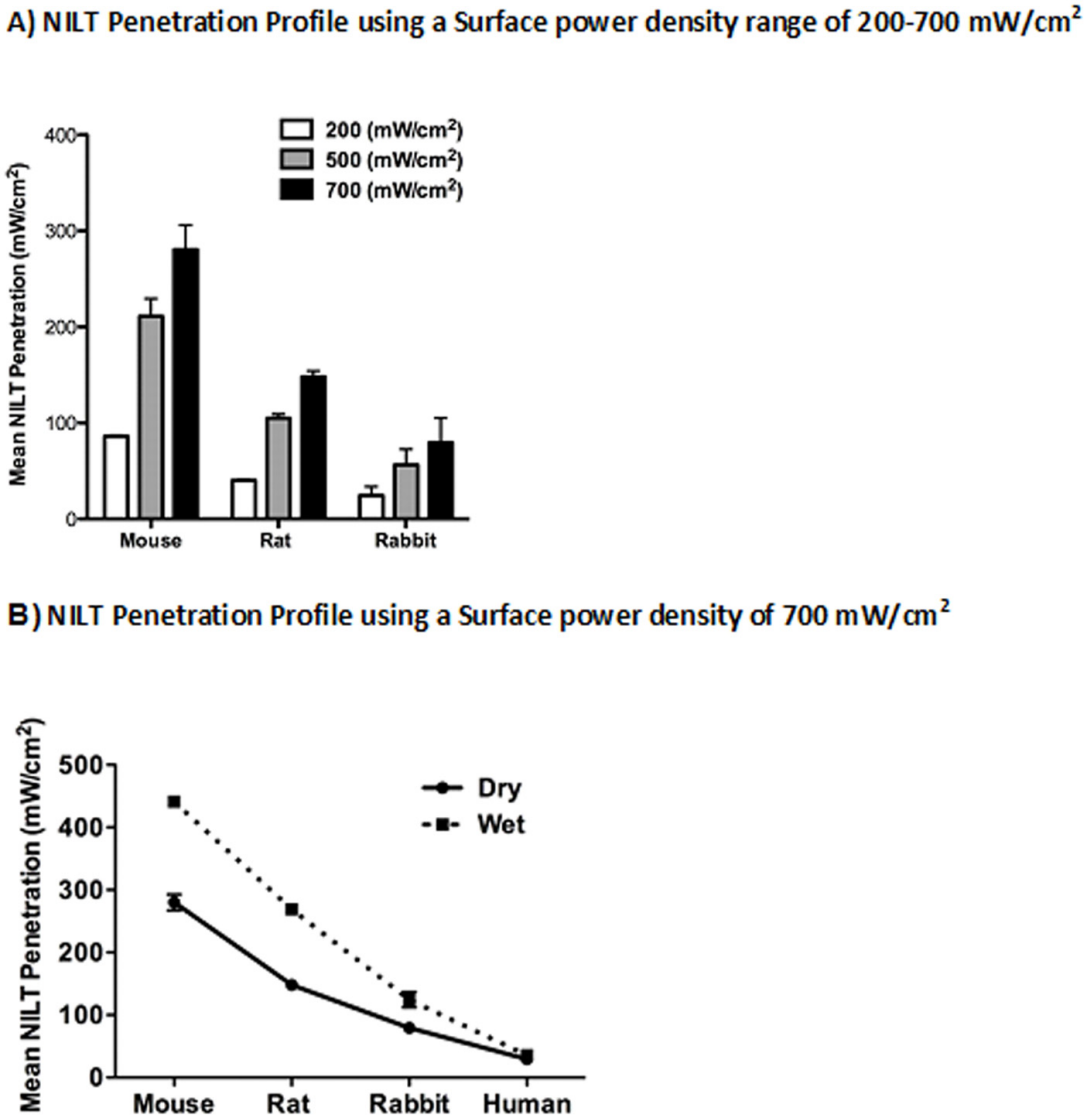
A) NILT Penetration Across the Skull of 3 Animal Species. Dehydrated and Hydrated Skull Analysis using a ***Power density of 200–700 mW/cm*** ^***2***^- NILT penetration through the skulls of all animal species utilized in this study. Presented as penetration (mW/cm^2^), calculated based on the area of illumination and the amount of power. In all 3 animals, there was increased NILT penetration through the hydrated (wet) skull compared to the dehydrated (dry) skull (*p<0.05). **B) NILT Penetration Across the Skull of 4 Animal Species:** Dehydrated and Hydrated Skull Analysis using a ***Power density of 700 mW/cm*** ^***2***^- NILT penetration through the skulls of all 4 species utilized in this study. Presented as penetration (mW/cm^2^), calculated based on the area of illumination and the amount of power. In all 3 animals, there was increased NILT transmission through the hydrated (wet) skull compared to the dehydrated (dry) skull (*p<0.05), but not with human calvaria.

In the study, we explored the penetration characteristics of 3 incrementally increasing power densities 200-500-700 mW/cm^2^ and showed that penetration is almost constant with the power density for each of the 4 species measured at bregma as power density increased (See Fig 6). In Table 1, it is important to note that we present skull thickness (in mm) for the 3 animal species used in this study. As skull thickness increases from 0.44 mm in mouse to 0.83 mm in rat to 2.11 mm in rabbit, NILT (mW/cm^2^) decreased from 40.10% in mouse to 11.36% in rabbit when dehydrated dry skulls were studied. In studies using hydrated and degassed skulls, skulls were submerged in deionized water for 48 hours while under vacuum to remove gases. When hydrated skulls were used for measurement, while there was no statistically significant change in thickness (p>0.05), we measured statistically significant increased NILT (56.6–81.5%). Moreover, there is a non-linear inverse relationship between NILT and skull thickness. Fig 6A shows the correlation between Mean NILT (% of power density in mW/cm^2^) as a function of power density. Moreover, in Fig 6B, we present data to show that there an incremental decrease in penetration that is inversely correlated with skull thickness (Fig 6B) when are 4 species are studied using a surface power density of 700 mW/cm^2^.

**Table 1:** Animal Skull Transmission: Comparison of Dehydrated- Hydrated State (Surface power density: 700 mW/cm^2^).

Species	Skull Thickness (mm)		NILT (mW/cm^2^)	
**A) MOUSE**	**Dry**	**Wet**	**Dry**	**Wet**
Mouse 1	0.41	0.41	305.72	441.30
Mouse 2	0.47	0.45	269.04	423.17
Mouse 3	0.46	0.47	250.70	459.44
Mouse 4	0.40	0.39	297.57	441.30
**Mean**	**0.44 ± 0.01**	**0.43 ± 0.01**	**280.76 ± 6.35**	**441.30 ± 3.70** *
**Percent Transmission**			**40.10**	**63.04** *
**B) RAT**	**Dry**	**Wet**	**Dry**	**Wet**
Rat 1	0.77	0.77	144.70	270.42
Rat 2	0.90	0.89	158.01	279.73
Rat 3	0.81	0.90	145.31	261.96
Rat 4	0.81	0.73	146.28	267.54
**Mean**	**0.83 ± 0.02**	**0.82 ± 0.04**	**148.73 ± 1.55**	**269.91 ± 1.85** *
**Percent Transmission**			**21.24**	**38.55** *
**B) RABBIT**	**Dry**	**Wet**	**Dry**	**Wet**
Rabbit 1	2.33	2.28	60.48	98.54
Rabbit 2	2.37	2.20	60.67	91.66
Rabbit 3	1.60	1.71	75.44	147.31
Rabbit 4	2.07	2.33	122.55	151.22
Rabbit 5	2.19	2.17	78.45	138.00
**Mean**	**2.11 ± 0.30**	**2.14 ± 0.11**	**79.52 ± 5.08**	**124.55 ± 5.38** *
**Percent Transmission**			**11.36**	**17.79** *

Hydrated state measured in air after degassing. Dehydrated state measured in air. Thickness & Mean Power Applied are provided as Mean ± SEM.

*Significantly different p<0.05. Mouse dia 2.5mm, Rat/Rabbit dia 11.7mm.

#### Human Skull Studies

In Table 2 we present the characteristics of the human skulls used in this study. We were able to include 9 males and 9 females with a median age of 73.61± 2.62 years; patients died of a wide variety of conditions including neurodegenerative diseases such as Alzheimer’s and Parkinson’s disease, cancer and lung disease. This Table is included so that we can document the diverse calvaria population that was studied; however, this study was not powered to determine the effects of gender or age on NILT penetration profiles.

**Table 2:** Human Skull Characteristics.

Calvaria ID #	Sex	Age	Cause of Death
10–258	M	61	Brain Metastasis, Cardiopulmonary arrest.
11–062	F	89	Congestive Heart failure
11–064	M	66	Prostate Cancer with bone metastasis
11–096	M	62	Pulmonary Fibrosis
11–264	F	71	Lung Cancer pulmonary failure
12–024	F	94	Atherosclerotic cardiovascular disease
12–028	F	80	Chronic obstructive pulmonary disease, Metastatic bladder carcinoma
12–047	M	80	Kidney Cancer
12–096	F	62	End stage Chronic obstructive pulmonary disease, Respiratory failure
12–160	M	72	Pneumonia, Chronic obstructive pulmonary disease, diabetes mellitus
12–223	F	77	Renal Failure, Metastatic cancer
12–320	M	79	Multi Organ Failure, Alzheimer’s disease, hypertension
**12–325**	**M**	**82**	**Respiratory failure, acute renal failure, Alzheimer's**
12–328	F	75	Dementia, Alzheimer's, Hypertension, Hyperlipidemia
12–330	M	60	Tumor lysis, metastatic lung cancer
12–357	F	53	Breast cancer, hepatic encephalopathy
12–360	F	86	Dementia, Parkinson's disease
13–008	M	76	Prostate cancer, Parkinson's disease

Tables 3 and 4 present cumulative data for NILT at 2 separate points measured for each skull, in order to determine if there are NILT differences within the same skull. In each Table, we focus on NILT (power density 700 mW/cm^2^) at bregma and a second point in the parietal region of the skull [26]. For correlation analysis of NILT penetration vs. skull thickness (mm) or radiological density (HU), we used CT scan data captured for either dehydrated skulls measured in air (Table 3) or hydrated and degassed skulls measured in air (Table 4). Fig 5 shows representative skull thickness and density profiles measured and constructed according to a recent study of Voie et al. [33]. Briefly, the panels in A show the exact coordinates for each measurement used in this study. The panel on the left is 3-D coordinate for bregma and on the right, for the parietal skull. The maps in B and C, which we will refer to as “heat maps”, show that each skull has a great deal of thickness and density variability over the entire surface of the skull.

**Table 3:** Human Calvaria NILT Profiles (Dry State- Air Scan): Surface power density 700 mW/cm^2^.

Calvaria ID #	Bregma Thickness (mm)	Bregma Density (HU)	Point 2 Thickness (mm)	Point 2 Density (HU)	NILT (mW/cm^2^)	
					Bregma	Point 2
10–258	7.12	1387.55	4.72	1223.69	25.41	37.13
11–062	8.52	1394.6	7.33	893.97	17.40	13.21
11–064	8.23	1313.30	6.59	989.15	3.54	11.54
11–096	7.25	1388.33	7.35	1200.86	28.76	29.50
11–264	5.75	1356.98	2.53	1074.03	58.25	63.75
12–024	6.69	1430.72	5.08	1210.02	33.04	35.64
12–028	6.42	1037.58	7.79	1057.16	20.29	18.24
12–047	8.14	1486.69	6.12	1238.25	25.03	43.64
12–096	6.48	1383.56	6.93	869.52	58.07	23.73
12–160	7.85	1250.59	5.24	881.57	34.15	35.83
12–223	6.06	1050.88	3.02	925.62	47.65	62.54
12–320	8.26	1482.81	8.33	913.11	27.45	19.82
***12–325***	***7*.*75***	***1337*.*56***	***5*.*13***	***1218*.*14***	***22*.*43***	***47*.*46***
12–328	9.33	1226.54	9.53	578.29	23.36	12.56
12–330	6.73	827.69	5.29	569.49	32.48	27.45
12–357	6.24	1127.77	6.59	746.17	5.96	0.65
12–360	6.09	1050.88	3.02	925.62	38.25	31.45
13–008	6.66	1137.58	5.90	713.13	25.78	20.85
Mean	7.19 ± 0.23	1259.53 ± 43.28	5.91 ± 0.44	957.10 ± 50.65	29.29 ± 0.81	29.72 ± 0.92
Mean Percent Transmission					4.18	4.24

Measures are provided as Mean ± SEM

**Table 4:** Human Calvaria NILT Profiles (Wet Degassed in Air): Surface power density 700 mW/cm^2^.

Calvaria ID #	Bregma Thickness (mm)	Bregma Density (HU)	Point 2 Thickness (mm)	Point 2 Density (HU)	NILT Transmission (mW/cm^2^)	
					Bregma	Point 2
10–258	7.08	1436.01	4.42	1321.83	29.03	34.25
11–062	8.53	1411.60	7.26	1015.48	24.94	17.22
11–064	7.88	1386.57	7.10	1054.29	17.77	42.99
11–096	7.47	1480.67	7.31	1288.73	34.06	29.87
11–264	5.20	1345.44	3.18	1023.46	58.63	61.88
12–024	6.72	1398.39	5.20	1241.66	33.13	33.97
12–028	6.83	1096.75	8.41	1103.20	21.59	20.29
12–047	7.60	1521.28	6.15	1218.38	26.80	42.43
12–096	6.40	1453.64	6.76	902.03	45.32	27.45
12–160	7.81	1443.83	7.88	1440.02	37.69	28.48
12–223	6.17	1043.14	3.22	1078.38	48.76	62.07
12–320	8.23	1533.98	8.09	971.40	30.71	14.33
***12–325***	***7*.*81***	***1420*.*37***	***5*.*15***	***1297*.*16***	***29*.*31***	***51*.*28***
12–328	9.43	1309.20	9.71	611.58	21.31	11.82
12–330	6.74	936.79	5.64	668.36	35.08	30.62
12–357	6.37	1217.87	6.70	886.95	26.34	8.65
12–360	6.19	1453.93	4.63	1140.81	42.43	34.43
13–008	8.59	1411.82	7.78	1246.99	34.43	30.99
Mean	7.28 ± 0.25	1350.07 ± 39.70	6.36 ± 0.42	1083.93 ± 52.19	33.19 ± 0.57	32.39 ± 0.83
Mean Percent Transmission					4.74	4.63

Measures are provided as Mean ± SEM

Tables 3 and 4 show that both thickness and density at bregma vary for each skull measured. Statistical analysis of thickness and density at both bregma and Point 2, for the entire group of skulls showed that there was no difference (p>0.05) between means, indicating no significant effect of hydration state on skull density or thickness. NILT penetration for dehydrated and hydrated skulls were not significantly different (p>0.05). Measures on both groups of skulls showed that 4.18–4.74% of NILT penetrates the skull at bregma, and there was no significant difference at parietal point 2.

**Fig 7:**
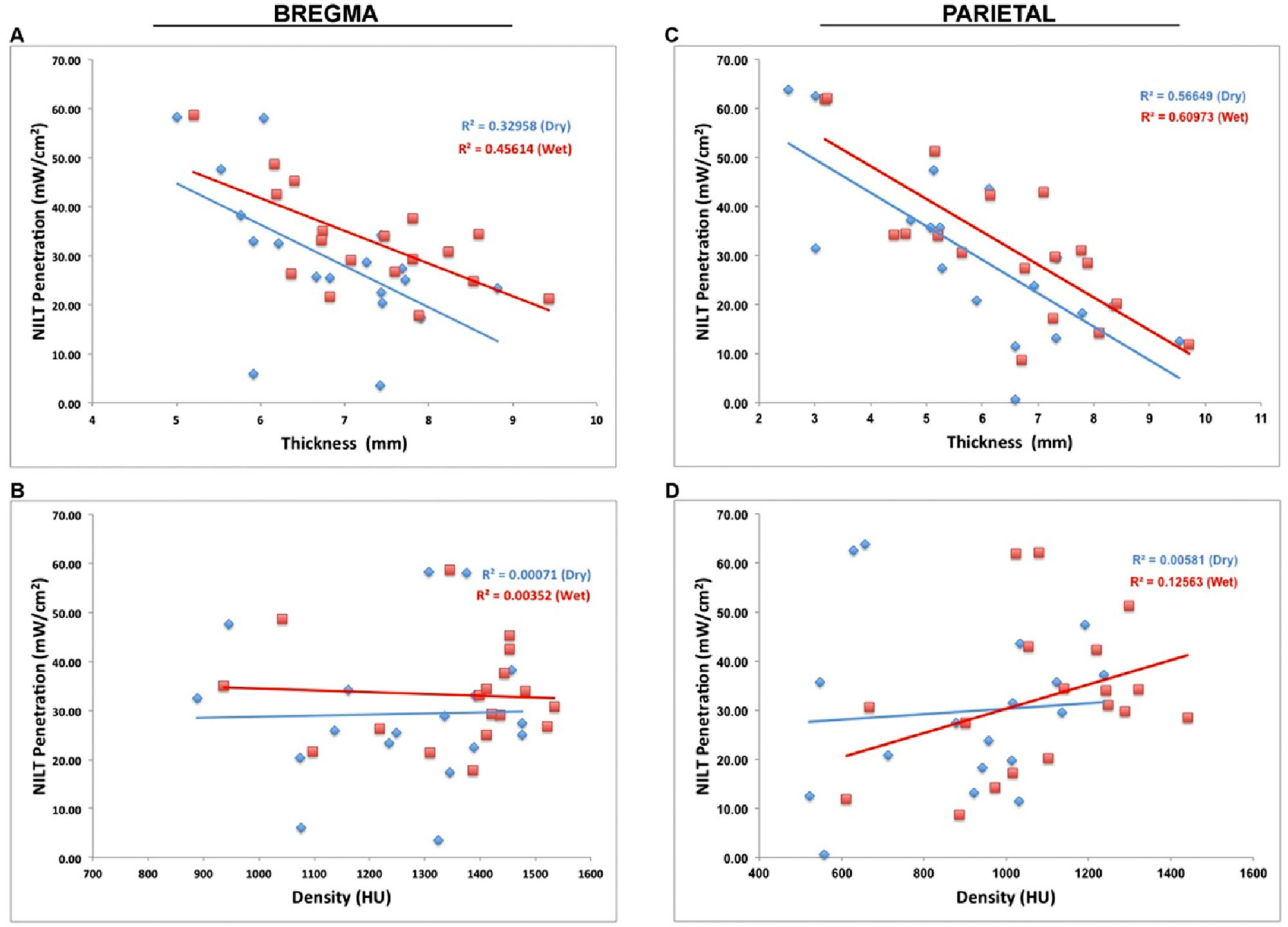
Correlation analysis: Human Calvaria- Thickness & Density. Plots of NILT penetration (mW/cm^2^) vs. thickness (mm) or density (HU). For each of the panels A-D, Pearson correlations are provided. Significant correlation between NILT penetration and thickness (p<0.05), but not density (p>0.05).

We determined if there was a correlation between NILT and thickness or density at bregma and the parietal skull for both hydrated (wet) and dehydrated (dry) skull states. As shown in Fig 7, we plotted absolute NILT penetration (mW/cm^2^) vs. thickness (mm) or density (HU) and used correlation analysis to calculate R^2^ values for each measurement point. The correlation coefficients are presented in each panel of the Figure.

In Panel A, the R^2^ values and (Pearson correlations) were 0.32598 (-0.57409, p = 0.0127) and 0.45614 (-0.67538, p = 0.0021) for dehydrated and hydrated skulls, respectively for thickness at bregma. In Panel C, the R^2^ values and (Pearson correlations) were 0.56649 (-0.75265, p = 0.0003) and 0.60973 (-0.78085, p = 0.0001) for dehydrated and hydrated, respectively for thickness at the parietal point.

In Panels B, the R^2^ values and (Pearson correlations) were 0.00071 (0.02658, p = 0.9166) and 0.00352 (-0.05933, p = 0.8151) for dehydrated and hydrated, respectively for density at bregma. In panel D, the R^2^ values and (Pearson correlations) were 0.00581 (0.07624, p = 0.7637) and 0.12563 (0.35444, p = 0.149) for dehydrated and hydrated, respectively for density at the parietal point. The correlation analysis demonstrates that there is a significant correlation between NILT penetration and skull thickness, whether the skull be dehydrated or hydrated (wet or dry), but there is no correlation between NILT penetration and skull density.

## Discussion

TLT is considered to be a non-invasive, leading edge technology that could have a huge impact on the way that neurodegenerative diseases are treated [3, 8, 19–21, 24, 34, 35], in particular AIS, TBI, AD and PD. It has been demonstrated that irradiation with specific wavelengths (nm) of infrared light (i.e. 800-808nm), allows for light penetration through the scalp, and to some extent the skull and brain tissues [13, 28, 29, 36]. The photobiostimulation, neuroprotective and clinical improvement effects of TLT are dep*e*ndent upon the quantity of laser light that can cross the skull as a barrier[15, 28, 29, 37–39], but a systematic approach and study to determine the extent of NILT penetration across the skull barrier of four commons research species has not been conducted.

In the present study, we found incremental attenuation of NILT penetration across the skull of 4 different species as skull thickness increased. Using a standard surface power density of 700 mW/cm^2^ across all 4 species, we found that there was greater than 95% attenuation of NILT penetrating the human skull; approximately 4.5% of NILT penetrated the human skull using dehydrated human calvaria (4.18–4.24%) and this attenuation was independent of hydration state (4.63–4.74% with hydrated degassed skulls). This is to be compared to 11.26% (Mean thickness 2.11 mm) in rabbits, 21.24% in rats (Mean thickness 0.829 mm) and 40.10% in mice, when measures were done on dehydrate skulls. This data indicates that NILT penetration significantly decreases as skull thickness increases. This level of penetration in humans skulls is similar to that reported by Haeussinger et al in a neuroimaging simulation article [40].

With animal skulls, there was also a significant effect of hydration state on NILT penetration: penetration in all 3 species significantly increased by 56.6–81.5% when the skull was hydrated and degassed to remove gaseous air pockets from the skull. With human calvaria, there was no significant effect of hydration state on NILT penetration. The correlation between decreased NILT penetration and skull thickness is extremely important considering the use of small animals for translational research [3, 4, 24–27, 29, 38, 39].

Because of the significance of TLT to treat neurodegenerative disease non-invasively, and more importantly, in order to correctly design future TLT animal studies, we will focus this section on the data obtained from human skulls. Using dehydrated human calvaria (Mean thickness 5.91–7.19 mm), with a power density of 700 mW/cm^2^, approximately 4.18–4.24% of laser light penetrated the dry skull. Thus, the power density ventral to the skull surface is estimated to be 29 mW/cm^2^, which is above the range of that previously shown to be beneficial in animal studies (10–25 mW/cm^2^, reviewed in [3, 4, 24, 26]), making the assumption that there are no additional barriers to penetrate. However, this finding should be considered and addressed with respect to the series of compartmentalized barriers that NILT was penetrate to produce a biological effect. Calvaria thickness calculations derived from CT scans for the mean skull thickness in humans is 6-7mm (this study). The documented thickness of the scalp is 5-6mm[41] and other major barrier spaces (the periosteum, the dura mater, subdural space and subarachnoid space) are approximately 3–4 mm [1]. Thus, the total barrier distance for NILT penetration is in the range of 14-17mm. If there is incremental attenuation of NILT penetration due to the additional barriers, even by 50–90%, then the delivered power density on the cortical surface would be estimated to be 2.9–14.5 mW/cm^2^. This would be in agreement with the abstracted conference proceedings study of Lychagov [32].

Taken together, we hypothesize that with the penetration barriers and the manual placement of the TLT probe at 20 pre-specified positions around the human head to deliver 8.3mW/cm^2^ for 2 minutes per treatment area, there may be insufficient penetration and coverage of the cortex and structures underlying the cortex. Thus, in effect, TLT will not effectively reach all cortical layers and subcortical structures damaged by ischemia or other disease processes. In essence, there will be limited activation of mechanisms shown to be important in neuroprotection and regeneration [3,4,12–14]. This may be viewed as a critical limitation of the treatment regimen design used in the NEST trials and the way in which TLT was being developed for delivery to patients.

There are a number of caveats in our study that should be raised. First, we chose to use an optimized penetration assembly system to systematically study NILT penetration in all 4 species. This included skulls and calvaria that were “dehydrated”, or “hydrated”. At least in animals, we showed that the dehydrated state allowed for less penetration of NILT due to the absence of water (i.e.: significantly increased penetration when skulls were tested in the degassed hydrated state). In humans, there was no significant differential effect when hydrated calvaria were compared to dehydrated calvaria. Moreover, the studies were done in the absence of overlying tissues such as skin of the scalp, periosteum, and underlying tissue such as dura mater (periosteal and meningeal layers), subarachnoid space and pia mater, all of which would further affect NILT penetration, and increase scatter or reflection [1].

The key observation made in this systematic study is that there is a significant correlation between NILT and thickness, and this is an important finding. Both between different calvaria used in this study (Tables 3 and 4), and within individual calvaria (Fig 5), we show extensive variability in thickness. Since we were able to demonstrate a significant correlation between decreased NILT and increased thickness of different calvaria, we can further extrapolate the data to individual calvaria. We who that is differential NILT in individual calvaria due to the heterogeneity of thickness (See Table 3, compare bregma with parietal for individual cavlaria).

In conclusion, our results demonstrate substantially different laser light penetration characteristics when all 4 species are compared directly. The study suggests that the use of small animal species with “thin” skulls, compared to humans, have important limitations. Our data suggests that small animals, while important for neurodegenerative disease research, cannot easily be used to develop TLT because of differential NILT penetration profiles and even scatter patterns (compare rabbit to human) across the skull. Efficacy in thin-skulled animals may overestimate TLT efficacy, leading to poor clinical trial design and failure in patients. The human scalp, skull and brain represent a significant barrier that must be overcome if TLT efficacy is to be achieved in blinded and randomized clinical trials. Because TLT has now been shown to be safe in rodents [29], rabbits [12, 13] and humans [3, 15, 16] under the specific treatment regimen used in translational studies and clinical trials (trials were safe at 8.3mW/cm2 for 2 minutes per treatment area), TLT neuroprotection should be further developed using an “***optimized treatment regimen***” that should take into consideration the architecture, anatomy and substantial barriers associated with the human scalp, skull and brain. This can be accomplished in animals using novel approaches incorporating the diverse data contained in this report and human skull characteristics.
